# Optimizing coolant loop design across the stator core: A research study

**DOI:** 10.1016/j.heliyon.2024.e36865

**Published:** 2024-08-24

**Authors:** Tian Xia, Chi Zhang, Hongjun Li

**Affiliations:** a Wuhan Institute of Shipbuilding Technology, Wuhan 430050, China; b School of Mechanical Engineering and Automation, Wuhan Textile University, Wuhan 430200, China; c Hubei Key Laboratory of Digital Textile Equipment, Wuhan Textile University, Wuhan 430200, China

**Keywords:** Permanent magnetic motor, Heat dissipation, Coolant channel, Stator yoke

## Abstract

Liquid cooling is widely used on high power motors. Optimal design of the coolant loop could help rise the power density. The coolant channels are placed across the stator core in this research. The heat dissipation performances of the coolant channel with different designs are compared and analyzed through simulation. Experiments were carried out to verify the simulation results and the feasibility of the cooling method. The results show that the coolant loop should be placed tightly close to the slots. The heat dissipation performance of the optimized coolant loop is efficient compared to the jacket cooling design. The coolant channels across the stator core could remain sealed under a 0.5 Mpa pressure at least according to the air proof test. The coolant pressure within the channels could be reduced efficiently through increasing the parallel loop number or moving the channels away from the slots properly. The winding temperature of the measured values and the simulated results of the motor with jacket cooling is within 2 °C.

## 1 Introduction

Liquid cooling is always preferred in high power motor due to its efficient performance on heat dissipation, including higher heat density, low noise and a separated system [1]. The design of the coolant circuit has a direct influence on the heat dissipation performance [2]. Jacket cooling is quite popular with commercial motor. But two thirds of the heat is generated by the winding and the stator teeth [3], the recycled coolant is far away from the slot with jacket cooling, the temperature rise of the winding can not be suppressed efficiently. Hence, the coolant channels are placed within the slots directly in some studies [4]. Remarkable heat dissipation performance of this method has been proved [5]. The winding temperature decreases more than 50 °C compared with the indirect cooling method [6]. But in actual production, high power motors are often designed with a large slot number for achieving excellent magnetic performance. Hence the slots are always narrow in circumferential direction. There is no enough space for the coolant ducts. Take the motor of BMW i3 for instance, the duct width was only 0.5 mm [7]. It brings excessive coolant pressure as a result of the high frictional loss within the ducts, the fabrication also becomes complex and expensive.

In this study, the coolant channels will be placed within the stator yoke in axial direction. The gap between the stator laminations was filled with insulation paint, so that the coolant leakage could be prevented without duct wall. Furthermore, the channels across the stator yoke brought direct impacts on both magnetic performance and cooling performance of the motor. It was hard to select the optimal channel design. Hence the channel design method in previous research of this study was applied [8]. The motors with the same magnetic performance but different channel designs were simulated and compared. The impacts of the channels on the magnetic performance and cooling performance will be decoupled, then the optimal coolant channel design could be found conveniently.

## 2 Method for simulation

### 2.1 Configurations of motor and cooling loop

**Fig. 1:**
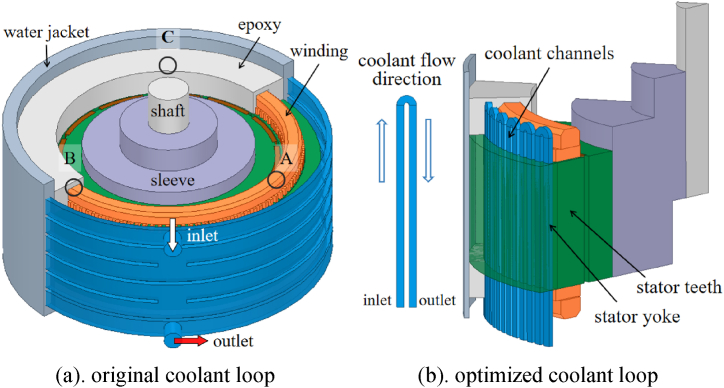
Sketch of the original and optimized coolant loop structures.

A PM (permanent magnet) motor with rated power 100 kW and rated speed 3000 rpm was applied in this study. Water jacket is used as the main heat dissipating method in original design as showed in Fig. 1a. The main dimensions of the motor are showed in Table 1. For improving the cooling performance of the motor, the coolant channels are placed across the stator core in axial direction, there is a one-to-one match from each slot to the coolant channel, hence the channel number is also 108 which is equal to the slot. Adjacent channels are jointed as a coolant loop. The length of each coolant loop could be adjusted according to the working condition. For convenience of analysis, the coolant loops with different length will be simulated and discussed. There is no metal duct within the stator core, the gaps between the stator laminations are filled with insulation paint through VPI (Vacuum Pressure Impregnation) process to prevent the coolant leakage. The metal ducts outside the stator core are fastened by the epoxy. As the motor structure is rotational symmetry, one-sixth of the motor which contains two pole pitches is considered in simulation. The related details are showed in Fig. 1b.

**Table 1:** Dimensions of the motor Unit:mm.

Stator diameter		Slot		Length		Thickness of water jacket	Pole pairs
Inside	Outside	Number	Width	Iron core	Motor shell		
275	364	108	4	100	190	17	6

### 2.2 Decoupling between heat dissipation and magnetic performance

**Fig. 2:**
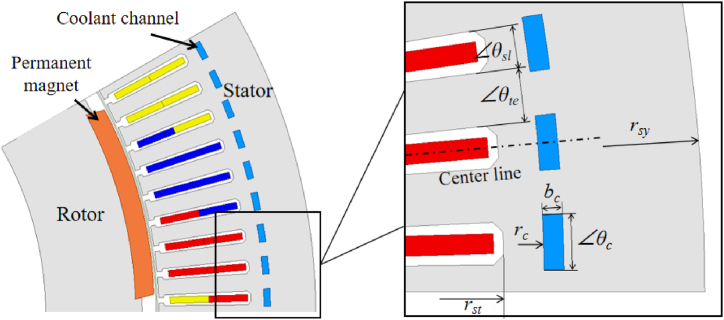
Design of the coolant channels.

The coolant channels are defined as illustrated in Fig. 2. The values of the loss were solved through the electromagnetic simulation by Maxwell. The core loss of the motor is divided into two parts: stator teeth and stator yoke (showed in Fig. 1b). So that the temperature distribution within the motor could be solved more precisely. The location and dimension of the channels depend on the value of parameter *k* and *l* as expressed in the following equations (eqs (1), (2), (3)):

(1) $$b_{c}=k\left(r_{sy}-r_{st}\right)\;\left(0<k<1\right)$$

(2) $$r_{c}=l\left(r_{sy}-r_{st}\right)+r_{st}\;\left(0<l<1-k\right)$$

(3) $$\theta_{c}=\frac{l\theta_{te}}{1-k}+\theta_{sl}$$

With this method, the change of the back EMF (Electric Motive Force) only depends on the channel height *b*_*c*_. After parameter *k* is defined, all the coolant channel designs at different locations *r*_*c*_ (parameter *l*) have the same impact on the back EMF of the PM motor as showed in Table 2. Furthermore, the stator core loss with different cases can also be calculated directly and approximately by equation (4). It can be concluded that the impact of the coolant channels on the electromagnetic performance and dissipation performance of the motor are completely decoupled with this design method. The comparison of the heat dissipation performance between different channel designs could be carried efficiently with the similar electromagnetic performance.

(4) $$P_{l}=P_{origin}+\frac{lb_{c}}{\left(1-k\right)l_{t}}P_{teeth}$$

**Table 2:** Electromagnetic performance of the motor with different channel designs.

Case	Location *l*	Height *k*	Back EMF (V)	Stator Core loss (W)	
				teeth	yoke
1	0.05	0.2	452.91	619.12	699.68
2	0.2	0.2	453.33	638.03	669.57
3	0.35	0.2	453.47	646.02	650.38
4	0.5	0.2	453.56	651	634.1
5	0.65	0.2	453.66	656.04	617.86
6	Original	design	453.68	671.76	590.94

**Fig. 3:**
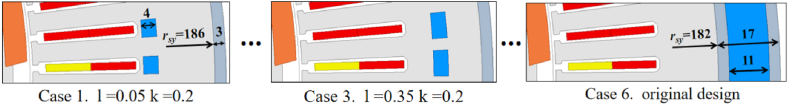
Design of the coolant channel in different cases.

*P*_*origin*_— stator core loss of original motor (W);

*P*_*teeth*_— teeth core loss of original motor (W);

*P*_*l*_— stator core loss with location *l* (W);

*l*_*t*_— length of the stator teeth (m);

Fig. 3 illustrates the location and shape of the coolant channels in different cases in Table 2. Compared to the jacket cooling, the coolant channels are moved into the stator core from the motor shell, meanwhile, the height of the stator yoke increases a height of the coolant channel 4 mm, but the shell thickness is also reduced by 14 mm, hence the outside diameter of the motor could be reduced about 20 mm compare with the original design. For keeping the back EMF constant, the cross area of the coolant channels rises obviously while the *l* increases according to eq (3). Since further increase of *l* will weaken the structural strength of the stator core significantly, the maximum value of location *l* in simulation is set as 0.65.

When the coolant flux is constant, the channel height has a greater influence on the coolant pressure rather than the motor temperature [9,10]. Hence, the study will be focused on the impacts of the channel location *r*_*c*_ and the coolant flux on the cooling performance. Furthermore, since the channels with small cross area may bring significant coolant pressure drop, the related influence factors including channel location and coolant flux will also be analyzed.

### 2.3 Simulation method

k-ε model was used to describe the flow of the fluids including the water and the air inside the motor. The energy transport equation applied in the simulation has the following form (eq (5)):

(5) $$\frac{\partial }{\partial t}\left(\rho h\right)+\nabla \cdot \left(v\rho h\right)=\nabla \cdot \left(k\nabla T\right)+S_{h}$$

*ρ*一density (kg/m^3^)

*h*一sensible enthalpy

*k*一conductivity (W/mK)

*T*一temperature (K)

*S*_*h*_一volumetric heat source (W/m^3^)

The stator core loss in Table 2 are used to define the *S*_*h*_ of stator teeth and yoke. While the *S*_*h*_ of winding is calculated by the rated current 187A.

### 2.4 Parameters used in simulation

The main thermal conductivity coefficients are reported in Table 3. The measuring method of the epoxy follows IO-10-87 (ASTM C518). The data is provided by the supplier ELANTAS company. The equivalent thermal conductivity of the stator core in axial direction is calculated using the method proposed by Zhang [11] with the stack coefficient 0.96. The data of slot winding are solved according to the copper space factor 0.43. The equivalent thermal conductivity of slot winding in the three directions are calculated using the empirical equation proposed by Wei [12].

**Table 3:** Thermal conductivity coefficients applied in simulation.

Region	Material	Thermal Conductivity (W/m·K)		
		Axial direction	Radial direction	Tangential direction
Stator core	35W270	0.62	32	32
Slot winding	About 43 % copper 57 % epoxy	171	1.75	1.75
epoxy	MC 622 LV/W 360	1.05	1.05	1.05
End winding	About 65 % copper 35 % epoxy	2.84	2.84	2.84
Shaft	Steel	42	42	42
Shell (Al)	Aluminum	237	237	237
Magnet	Nd-Fe-B	7.7	7.7	7.7

## 3 Air proof test

**Fig. 4:**
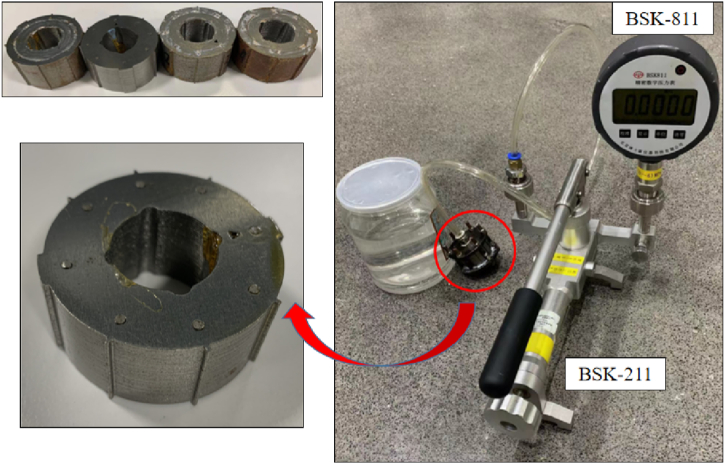
Apparatus applied in the test.

**Fig. 5:**
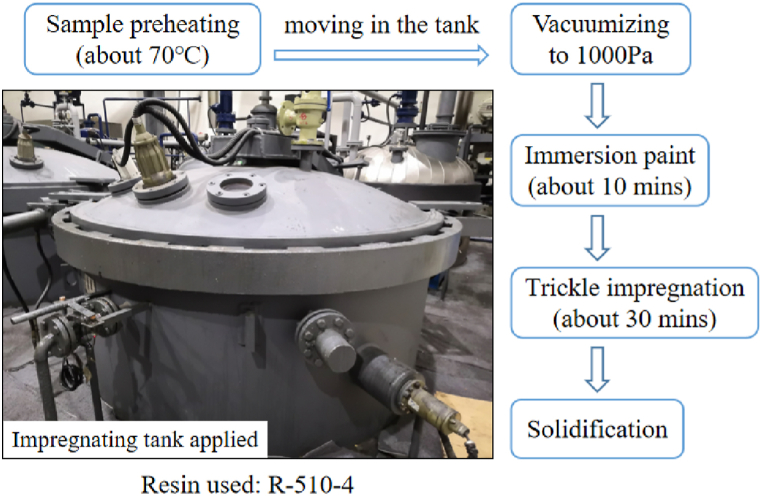
Main procedure of the VPI process.

For validating the feasibility of the optimized coolant channel design, Air proof test was carried out to check the waterproof performance of the coolant loop. First, a type of simple iron core (outer diameter 46 mm) was designed and assembled as showed in Fig. 4. There was a hole with a diameter of 20 mm at the centre of the lamination. The thickness of each lamination is 0.5 mm. 40 pieces were used for each iron core. Then, VPI (Vacuum Pressure Impregnation) process was applied on the samples before experiment as illustrated in Fig. 5. The distance of the two metal covers remained the same along the circumferential direction through conducting the four nuts. As a result, the gaps between the layers of the core could be similar across the sample approximately. The bottom side of the core was sealed. The top side was connected with the air pump through a plastic duct as showed in Fig. 4.

The sample was then placed in the water, the air pressure 0.5 Mpa was applied. The waterproof performance was observed continuously for 10 min 4 samples were prepared for the test. For each sample, the test was carried out three times with the metal covers re-installed every time. So that the impact of the external pressure difference on the iron core on the air proof performance could be estimated.

## 4 Results and discussion

### 4.1 Influence of the loop design

**Fig. 6:**
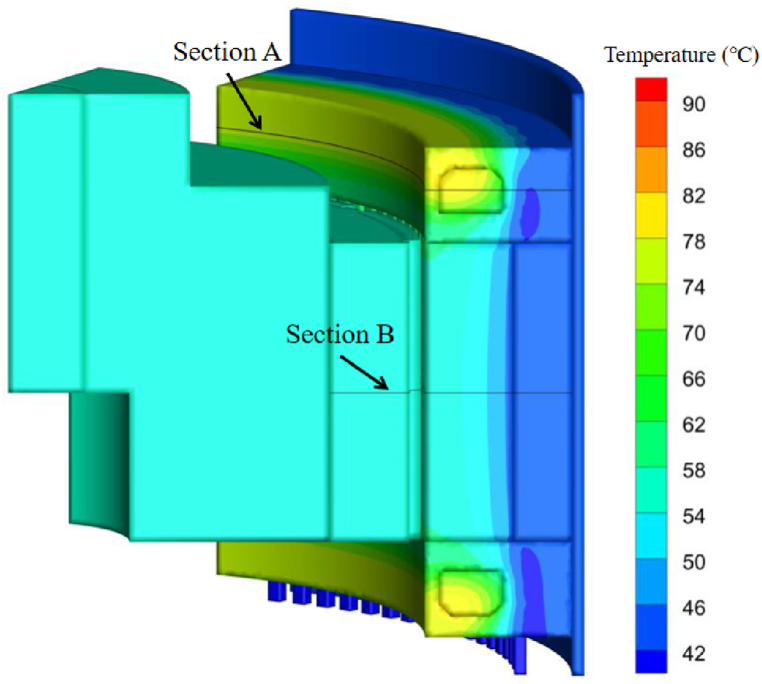
Temperature distribution with the coolant channels *l* = 0.05(Q = 48.6 L/min).

**Fig. 7:**
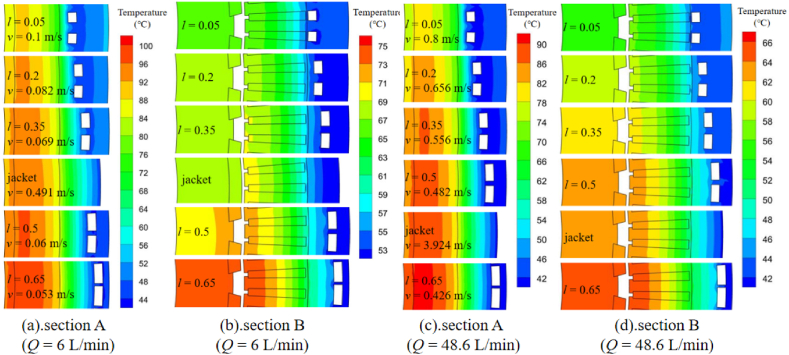
Temperature distributions of section A and B with different coolant fluxes *Q*.

Fig. 6 shows the temperature distribution of the motor with the coolant channels *l* = 0.05 (loop A in Fig. 10 is applied). It can be seen that the heat dissipation performance is efficient when the coolant flux is sufficient (48.6 L/min). The highest temperature appears around the end winding. The characteristics of the temperature distribution is similar to traditional inner rotor jacket cooling motor. For further study on the optimal cooling design, the temperature distributions with various coolant flux *Q* and parameter *l* are simulated for comparison. Since the temperature distributions on section A and B (showed in Fig. 6) are centrosymmetric approximately and respectively when loop A is applied, the results within a small random area are selected and showed in Fig. 7. As there is a little temperature difference in tangential direction with jacket cooling, the hottest area on section A and B (360° range) are selected for comparison respectively.

fig. 7aand b shows that when the coolant flux is not sufficient (6 L/min), the flow rate has obvious impact on the temperature distribution. The temperature of winding and stator core increases gradually while the coolant channels are moved away from the winding in radial direction (from *l* = 0.05 to *l* = 0.65). However, since the flow rate with jacket cooling is much higher than the other coolant loop designs due to its single loop structure (one inlet and one outlet). The winding temperature with jacket cooling is lower than the coolant channel *l* = 0.5, even if the distance between coolant and the winding is the farthest with jacket cooling. While the coolant flux rises to 48.6 L/min as showed in Fig. 7c and d, the winding temperature with jacket cooling increases relatively compared to the other designs. It is between the values with coolant channel *l* = 0.5 and *l* = 0.65. The results indicate that as the coolant flow rate rises, the impact of the flow rate on the motor temperature will be weaken, the cooling performance mainly depends on the distance between coolant and the winding.

**Fig. 8:**
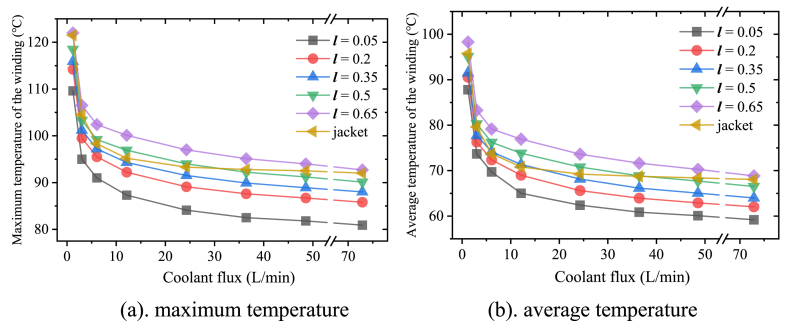
Change of the maximum and average winding temperature with the coolant flux.

Fig. 8 shows the winding temperature as a function of the coolant flux. It can be seen that the maximum temperature (Fig. 8a) and average temperature (Fig. 8b) of the winding fluctuate in a similar pattern. When the coolant flux is not sufficient (*Q* < 6 L/min), the winding temperature decreases quickly as the coolant flux rises. Then further improvement of the heat dissipation becomes more and more weak even great flux increase is applied (from 6 L/min to 73 L/min). The temperature difference between the different curves is also stable except the curve of jacket cooling. Since the flow rate with jacket cooling is already fast enough with coolant flux 12.15 L/min (0.98 m/s), the motor temperature almost remains unchanged with any flux above that. Therefore, according to the variation trend in Fig. 8, it can be deduced that the motor temperature with jacket cooling is still the highest when the coolant flux is great enough.

### 4.2 Influence of the loop length

**Fig. 9:**
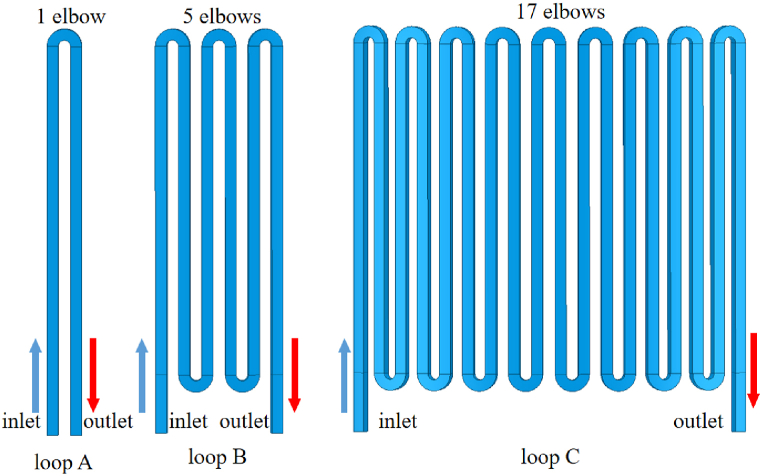
The structure of coolant loop with different length (*l* = 0.05).

It can be concluded from above that the coolant channels with *l* = 0.05 brought the best performance in suppressing the temperature rise. Furthermore, high flow rate of the coolant benefits the dissipation performance with the same flux. In practical production, excessive parallel coolant loops in one motor (54 with loop A in Fig. 7) make the motor structure complicated and lead extra trouble in loop connection. Hence, adjacent channels (*l* = 0.05) are connected as showed in Fig. 9, so that the coolant flow rate could be rose significantly with the same flux. Meanwhile, the number of the parallel loops could be also reduced greatly. For instance, from 54 to 6 when the loop C (with 17 elbows) is applied.

**Fig. 10:**
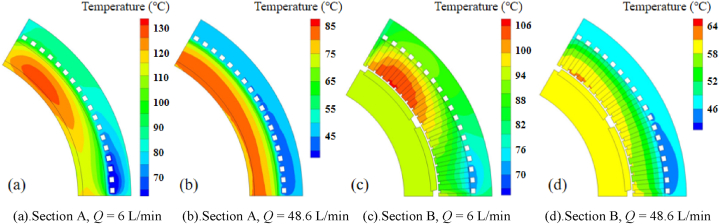
Temperature distributions of section A and B with different coolant fluxes *Q* (*l* = 0.05, loop C).

While the elbows of the loop increases, the motor temperature differs in tangential direction inevitably as showed in Fig. 10. When the coolant flux is 6 L/min, the temperature difference in tangential direction reaches at least 25 °C across the end winding (Fig. 10a) while 20 °C across the slot winding (Fig. 10c). The service life of the winding insulation near the high temperature region may get reduced significantly. Increasing the flux to 48.6 L/min, the temperature difference of the winding in tangential direction declines below 5 °C as showed in Fig. 10b and d.

**Fig. 11:**
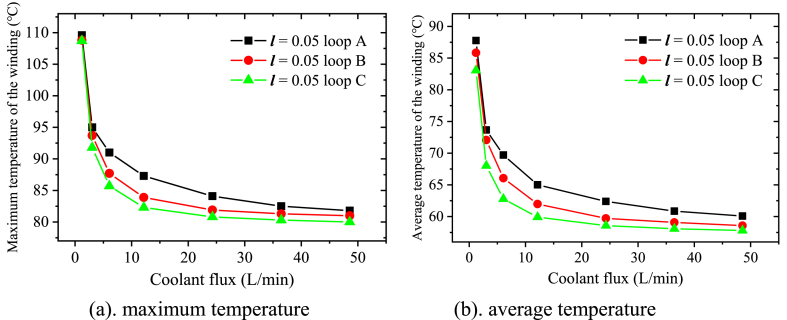
Change of the maximum and average winding temperature with different coolant loops.

Fig. 11 shows that obvious improvement of the dissipation performance could be made while the loop length increases. Compared with loop A, the maximum temperature of the winding is about 5.3 °C lower with the flux 6 L/min while loop C is applied (Fig. 11a). The decrease of the average temperature is greater (about 7 °C as showed in Fig. 11b). As the coolant flux increases, the impact of the flow rate gets weakened gradually, the winding temperature difference between loop A and loop C declines to about 2 °C (1.8 °C in Fig. 11a while 2.2 °C in Fig. 11b) with the flux 48.6 L/min.

### 4.3 Coolant pressure and the waterproof performance

The sectional area of the channel is smaller than that of the jacket cooling obviously. Greater friction loss along the channel is inevitable with the same coolant flux. The coolant pressure will rise as a result. The waterproof performance of the coolant loop gets to be a critical problem in practical application.

The coolant pressure along the channel could be expressed as eq (6) as followed [13]. It consists of three parts: friction loss *h*_*f*_, elbow loss *h*_*e*_ and the pressure related to the height difference along the loop *h*_*d*_. The fanning friction factor *f* is calculated according to eq (7), [14].

(6) $$\begin{array}{c} P_{t}=\rho g\left(h_{f}+h_{e}+h_{d}\right)\\ h_{f}=\frac{fv^{2}\left(\left(n+1\right)L_{c}+nL_{e}\right)}{2gD}\\ h_{e}=\frac{nk_{b}v^{2}}{2g}\\ D=\frac{4A}{C} \end{array}$$

(7) $$\frac{1}{\sqrt{f}}=-1.8\;\log\left[{\left(\frac{e/D}{3.7}\right)}^{1.11}+\frac{6.9}{\text{Re}}\right]$$

*ρ* — coolant density (kg/m^3^);

g — gravitational acceleration (m/s);

*L*_*c*_ — length of the stator core (m);

*L*_*e*_ — length of the elbow within the epoxy (m);

n— number of the elbows of each coolant loop (as showed in Fig. 9);

*f* — Fanning friction factor;

*v* — the flow rate of the coolant (m/s);

D— hydraulic diameter (m);

*k*_*b*_ — loss coefficient of the elbow;

A— the sectional area of the coolant channel (m^2^);

C— the perimeter of the cross profile of the coolant channel (m);

*e/D—* relative roughness;

*Re—* Reynolds number;

When the coolant loop with 17 elbows is applied. there are 6 parallel coolant loops within the motor. Despite the pressure related to the height difference along the loop, the coolant pressures are calculated and showed in Fig. 12. Since the sectional area of the channel decreases as the location *l* reduces, the flow rate *v* within the channels *l* = 0.05 increases the fastest while the coolant flux rises. As a result, the coolant pressure within the channels *l* = 0.05 is greater than the others obviously as showed in Fig. 12. According to the results in Fig. 11, the reduction of winding temperature is small relatively after the coolant flux exceeds 12 L/min. Therefore, a waterproofing pressure of 0.2–0.3 Mpa should be enough for the optimized channel design.

**Fig. 12:**
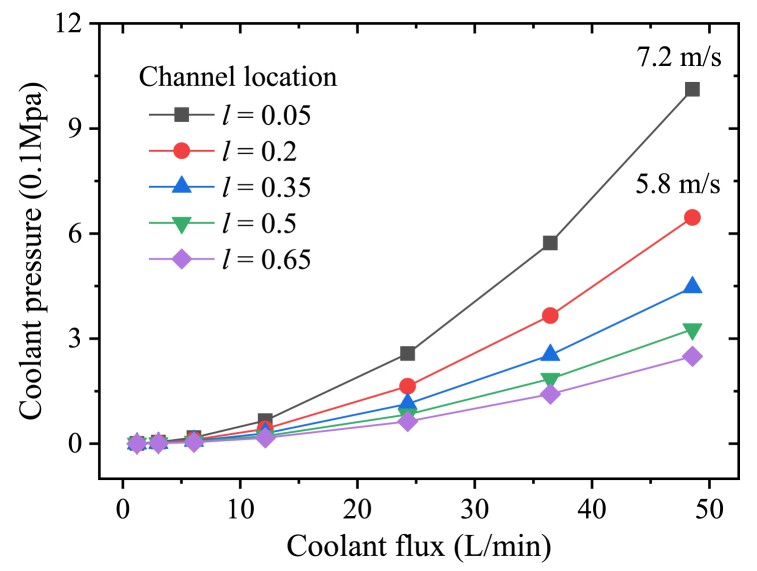
Coolant pressure with different channel locations (17 elbows).

It can be concluded that the heat dissipation performance with the coolant channels *l* = 0.05 is the most efficient, but its coolant pressure is also the highest. The coolant pressure could be controlled through conducting the loop length, the number of the parallel loop should be defined carefully according to the slot number. Furthermore, moving the coolant loop away from the slots (increasing *l*) appropriately is also an efficient method to suppress the coolant pressure, even if the winding temperature may get a little higher.

Table 4 shows the result of the air proof test. It is showed that the air proof performance of the samples is good and stable. Since the viscosity of water is far greater than the air, the maximum waterproof pressure of the sample is also at least 0.5 MPa. It can be concluded that the optimized liquid cooling method is practicable and can be widely used.

**Table 4:** Results of the air proof test.

Sample	Cover installation	Point of gas leaking	Initial pressure (MPa)	Pressure drop after 10 min (Kpa)
a	1	0	0.5068	1.6
	2	0	0.503	1
	3	0	0.5039	0.7
b	1	0	0.5018	0
	2	0	0.5029	1.2
	3	0	0.5016	1
c	1	0	0.508	0.8
	2	0	0.5058	1
	3	0	0.503	0.6
d	1	0	0.5034	1.6
	2	0	0.5077	1.5
	3	0	0.5094	0.9

### 4.4 Verification of the motor temperature

Three PT-1000 thermalcouples were applied in verification experiment of the motor with jacket cooling. The thermalcouples were embedded inside the end winding as showed in Fig. 1a (Point A, B and C). The probes were located along the circumferential direction with an interval of 120°. The positions of the three were rotational symmetric. After the installation, the end winding as well as the slots were filled up with the epoxy. Hence the locations of the thermalcouples were well fixed during experiment.

On the rated condition, the end winding temperature was measured with different coolant fluxes and coolant temperatures. Since the water duct between the motor and the water tank is about 3m, the coolant temperature could not be controlled accurately with low flux. Therefor the experiment was carried out with the coolant flux more than 6L/min. The data were recorded every 30 min till the temperature is constant.

**Fig. 13:**
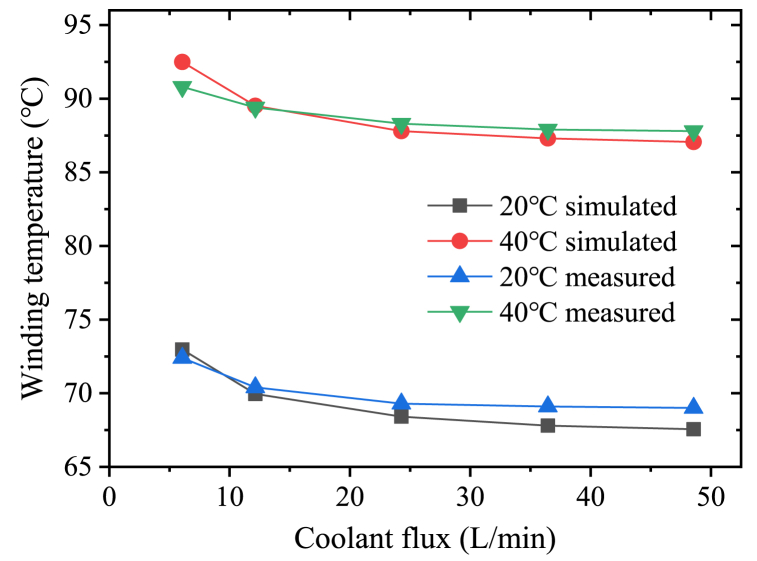
Comparison between the measured temperatures and the simulation results.

Fig. 13 compares the measured temperatures with the simulation results. The measured temperature of the winding is the average value of the three thermalcouples (A, B, C in Fig. 1a). The differences between the measured temperatures and the simulations result are within 2 °C. It can be concluded that the parameters of the materials and the boundary conditions used in the models are appropriate.

## 5 Conclusions

For improving the heat dissipation performance of the motor with liquid cooling, the coolant loop across the stator yoke was applied on the motor instead of the jacket cooling. The impacts of the coolant loops on the heat dissipation and magnetic performance were decoupled with appropriate channel design. As a result, the correlations between the channel design and the heat dissipation performance could be studied independently and correctly. In this study, the optimized coolant loop was compared with the jacket cooling through thermal simulation. The characteristic of the optimized coolant loop on the heat dissipation was analyzed carefully. The feasibility of the application of the optimized loop was also evaluated through the water proof test. The following conclusions could be made.

1. The optimized coolant channels should be placed tightly close to the slots for achieving the best heat dissipation performance. The winding temperature increased gradually while the channels were moved away from the slots in radial direction. When the channels were near the motor shell, the heat dissipation performance was similar to that of the jacket cooling.
2. The optimized coolant loop could remain sealed under 0.5 Mpa coolant pressure at least. But excessive coolant pressure increase might still occur in some cases. The pressure could be reduced efficiently through increasing the parallel loop number or moving the channels away from the slots properly.

## Data availability

The data associated with this study has not been deposited into a publicly available repository, and all relevant data was included in article/supplementary material/referenced in article.

## CRediT authorship contribution statement

**Tian Xia:** Writing – review & editing, Writing – original draft, Validation, Investigation, Funding acquisition, Formal analysis. **Chi Zhang:** Writing – review & editing, Resources. **Hongjun Li:** Writing – review & editing, Resources.

## Declaration of competing interest

The authors declare that they have no known competing financial interests or personal relationships that could have appeared to influence the work reported in this paper.
